# DoSDefender: A Kernel-Mode TCP DoS Prevention in Software-Defined Networking

**DOI:** 10.3390/s23125426

**Published:** 2023-06-08

**Authors:** Dongbin Wang, Yu Zhao, Hui Zhi, Dongzhe Wu, Weihan Zhuo, Yueming Lu, Xu Zhang

**Affiliations:** 1 School of Cyberspace Engineering, Beijing University of Posts and Telecommunications, Beijing 100876, China; 2 Engineering Research Center of Blockchain and Network Convergence Technology, Ministry of Education, Beijing 100876, China; 3 TravelSky Technology Limited, Beijing 100190, China; zhihui@travelsky.com.cn; 4 Cyberspace Security Research Center, Peng Cheng Laboratory, Shenzhen 518055, China; 5 Tencent, Shenzhen 518000, China; 6 Key Laboratory of Ministry of Education and Trustworthy Distributed Computing and Service, Beijing 100876, China; 7 National Engineering Research Center of Mobile Network, Beijing University of Posts and Telecommunications, Beijing 100876, China

**Keywords:** software-defined networking, denial of service, connection proxy

## Abstract

The limited computation resource of the centralized controller and communication bandwidth between the control and data planes become the bottleneck in forwarding the packets in Software-Defined Networking (SDN). Denial of Service (DoS) attacks based on Transmission Control Protocol (TCP) can exhaust the resources of the control plane and overload the infrastructure of SDN networks. To mitigate TCP DoS attacks, DoSDefender is proposed as an efficient kernel-mode TCP DoS prevention framework in the data plane for SDN. It can prevent TCP DoS attacks from entering SDN by verifying the validity of the attempts to establish a TCP connection from the source, migrating the connection, and relaying the packets between the source and the destination in kernel space. DoSDefender conforms to the de facto standard SDN protocol, the OpenFlow policy, which requires no additional devices and no modifications in the control plane. Experimental results show that DoSDefender can effectively prevent TCP DoS attacks in low computing consumption while maintaining low connection delay and high packet forwarding throughput.

## 1. Introduction

Software-Defined Networking (SDN) decouples the control plane from the data plane, which introduces a more flexible way to manage the network with fine-grained and centralized control [1]. The control plane is responsible for making forwarding path decisions, and the data plane is responsible for delivering the packets to the destination. As the de facto standard SDN protocol, OpenFlow introduces a reactive mechanism to handle the packets with the match-action paradigm. The data plane, Open vSwitch, handles the packets based on flow tables. When the incoming packet is not matched with the existing flow rules in the flow table (known as a table-miss), the data plane encapsulates the packet in a packet-in message and delivers it to the controller. After receiving the packet-in message, the controller computes and sends a forwarding path back to the switch with a flow-mod message. The switch installs it as a flow rule in the flow table and follows the flow rule to handle the packet.

The reactive packet handling mechanism and the limited processing capability of the centralized controller become a bottleneck in SDN. It leads to performance degradation and security problem in SDN. When a packet comes which is not matched with the existing flow rules in the flow table, the data plane sends a packet-in message to the control plane for the decisions to handle the packets. An attacker can leverage this costly communication to launch SDN-aimed Denial of Service (DoS) attacks based on Transmission Control Protocol (TCP) attacks [2,3,4]. Specifically, the attacker can generate a massive of forging packets, making them hard to match any existing flow rules on a victim switch. Then, a large amount of table-miss is incurred, and a massive packet-in message is sent to the controller, which consumes communication bandwidth, CPU, and memory in the control and data planes. TCP DoS attacks increase the delay of forwarding packets and degrade available bandwidth in SDN.

To protect SDN against TCP DoS attacks, the following two challenges should be solved:

- How to effectively prevent TCP DoS attacks from entering SDN?
- How to efficiently handle TCP DoS attacks while maintaining low computing consumption, low connection delay, and high packet forwarding throughput?

For the first challenge, TCP DoS attack traffic is mixed with normal traffic in SDN. It is difficult to distinguish TCP DoS attack traffic from normal traffic; therefore, TCP DoS attack traffic cannot be prevented from entering SDN. Packets that incur table-miss cannot be simply dropped because normal packets will be dropped as well. The genuine attempts to establish a connection should be distinguished from the bogus attempts, and the packets of the attack connection should be filtered out. DoS attacks incur massive table-miss, consume the resources of the control plane and the data plane, and degrade the performance of SDN. So the second challenge is how to handle TCP DoS attacks while maintaining a low-cost way and high packet forwarding throughput.

Some previous research work has been devoted to mitigating SDN-aimed DoS attacks. Avant-Guard, implemented into the software-based OpenFlow reference switch that has a slow userspace datapath, introduces a connection migration module on the data plane to identify attacks by verifying the handshake of each new TCP connection [5]. FloodDefender detours some table-miss packets from the victim switch to the neighbor switches and prevents the communication bandwidth from being exhausted between the victim switch and the controller [4]. FloodGuard introduces a data plane cache to protect the controller [6]. FloodShield maintains a global binding table in the control plane in which each binding entry binds a source address to validate the source address [7]. Unfortunately, there are no satisfactory solutions that can effectively prevent TCP DoS attacks from entering SDN with low computation cost, low connection delay, and high packet forwarding throughput.

In this paper, DoSDefender is proposed as a kernel-mode TCP DoS prevention framework in the data plane to protect SDN against TCP DoS attacks. All designs of DoSDefender conform to the de facto standard SDN protocol and OpenFlow policy, requiring no additional devices and no modification on the control plane. DoSDefender monitors traffic and detects TCP DoS attacks in the data plane. When DoS attacks are detected, DoSDefender begins to verify the validity of the attempts to establish a connection and relay all normal TCP data packets in kernel space between the source and the destination, as occurs during normal TCP sessions.

To prevent TCP flood, our main technical contributions are as follows:

- DoSDefender is proposed as a kernel-mode TCP DoS prevention framework to verify the validity of the attempts to establish a connection from the source address and prevent TCP DoS attacks from entering SDN.
- DoSDefender migrates the connection between the source and the destination in kernel space while avoiding the extra consumption in copying packets between kernel space and user space in handling packets and improving the performance in defending TCP DoS attacks.
- DoSDefender is designed and implemented in the data plane to prevent TCP DoS attacks. Experimental evaluation shows that DoSDefender can effectively prevent TCP DoS attacks and protect the computation resource in the control plane while maintaining low connection delay and high packet forwarding throughput.

The rest of the paper is organized as follows. Section 2 states some background knowledge and the security problem of SDN-aimed DoS attacks. The related work is summarized in Section 3. In Section 4, the detailed designs of DoSDefender are presented. The experimental evaluation of DoSDefender is shown in Section 5. Section 6 provides a conclusion.

## 2. Problem Statement

### 2.1. SDN Workflow

In SDN, the switch handles the packets based on the existing flow rules received from the controller. After receiving incoming packets, the packets will be matched with the flow rules in the switch. When no flow rules in the flow table match the incoming packet (known as a table-miss), the packet is encapsulated in a packet-in message and sent to the controller by the switch. After the controller receives the packet-in, it computes a forwarding path and installs it as a flow rule in the switch with the flow-mod message. This reactive flow installation approach in SDN provides a more flexible way to manage and process packet.

### 2.2. Adversary Model

**Figure 1:**
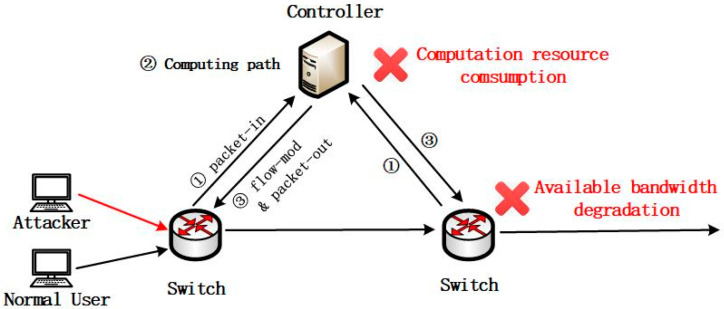
Denial of Service attack in SDN.

The reactive flow installation approach in SDN and the single point failure, limited computation resource of the centralized controller, could be leveraged by the attackers. TCP DoS attacks against SDN are depicted in Figure 1. The attacker generates a large massive of forged TCP packets with SYN, the first packet of a three-way handshake, which is mixed with the packets of the normal traffic and is hard to distinguish. The spoofed packets are matched with a low probability by any existing flow entries of the switch in the data plane, which incurs a massive table miss. The switch has to cache the packets and send a number of packet-in messages for unmatched packets to the controller. The controller has to consume a number of computation resources to compute the forwarding path and install it as a flow rule into the flow table in the switch with a flow-mod message.

The packet-in message and packet-out message incurred by TCP DoS attacks jam the bandwidth between the controller and the switch, exhaust the computation resource in the controller, increase the latency of forwarding packets, degrade the available bandwidth for forwarding packets, and overload the flow table in the switch by installing useless flow rules in a short time. TCP DoS attacks significantly degrade the performance of SDN.

## 3. Related Work

Software-Defined Networking, as the new network architecture, provides a global view of the network and centralized and fine-grained management of network flows. Due to single-point failure, limited computation resources in the control plane, and communication bandwidth between the control plane and the data planes, TCP DoS attacks are serious security problems in SDN. Several approaches in DoS detection methods and defense mechanisms are provided in recent research to prevent SDN-aimed TCP DoS attacks. However, each of them has some challenges in real-world scenarios.

### 3.1. Detection Methods

A series of DoS attack detection and mitigation methods, classifying DoS traffic and normal traffic, have been proposed. SAFETY uses an entropy-based detection mechanism to mitigate TCP SYN flooding attacks in SDN [8]. The ensemble learning techniques are introduced to cooperatively detect DoS attacks in SDN [9]. A generalized entropy (GE) is introduced to detect the low-rate DDoS attacks on the control plane [10]. However, the defined threshold value of the mechanisms based on entropy broadly depends on the security researchers’ experience. DoSGuard uses a support vector machine (SVM) as the classifier to detect and filter DoS attacks and protects networks [11]. A deep learning approach based on Gated Recurrent Unit (GRU) was introduced to handle DDoS attacks in SDN [12]. FloodDetector utilized two machine learning classifiers, K-nearest neighbor and artificial neural network, to detect both known and unknown flooding attacks [13]. SA-Detector framework uses self-similarity to detect the malicious OpenFlow traffic for a group of flooding attacks [14]. A generative adversarial network (GAN) framework is used to mitigate DoS attacks in SDN [15]. Deep learning techniques based on long short-term memory and Autoencoder are used to handle DoS attacks in SDN [16].

Although a series of DoS attack detection and mitigation methods have been proposed Recently, DoS has become more sophisticated and not easy to be identified. The false-positive rate affects the desirable detection accuracy and protection effect.

### 3.2. Defense Mechanisms

Various DoS defense mechanisms are proposed to protect SDN. Avant-Guard is implemented into the software-based OpenFlow reference switch that has a slow userspace datapath [5,17]. Avant-Guard introduces a connection migration module on the data plane to identify DoS attacks by verifying the TCP handshake of the connections [5]. However, the connection migration will inevitably copy data between user space and kernel space which will degrade the performance of the implementation. FloodGuard introduces a defense framework against DoS attacks to prevent overloading switches and control channel bandwidth and the controller [6]. It uses a proactive flow rule analyzer to preserve network policy enforcement and packet migration to protect the control plane from being overloaded. They handle all the incoming packets in place of the controller. However, the proposed approach may result in an enhanced delay in processing the data packets that increases the time of setting up the new rules. A security policy protocol and client authentication model were proposed to permit the authorized hosts to use the network resources that avoid unauthorized flooding attacks on SDN networks [18]. But the performance of SDN is compromised inevitably. FloodDefender stands between the control plane, and the apps are designed to protect the communication channel between the control plane and the data plane [19,20]. FloodDefender uses table-miss engineering to divert the table-miss packets to the neighbor switches, packet filtering to filter the one-packet flows from triggering the packet-in message, and flow table management to eliminate useless flow rules periodically. When a massive of table-miss packets are sent to the control plane, regardless of whether it is a forgery or not, the computation resources of the control plane will be exhausted. The effects of protection are inevitably compromised. A QoS-aware mitigation strategy is implemented to find the under-loaded switches in the network and control the table overflow attacks [21]. A framework based on histogram-based gradient boosting is proposed to detect and mitigate low-rate DoS attacks in SDN [22]. The finding peaks algorithm is used to find the attacker via peak properties of the flow and install flow rules on the switches to drop the attack packet and mitigate the DoS attacks. However, it also will drop the normal flows because of the identification accuracy.

Summarizing the above approaches, no satisfactory defense solution can effectively prevent TCP DoS attacks from entering SDN with low connection delay and high packet forwarding throughput. New schemes are needed to protect SDN against TCP DoS attacks at a low cost.

## 4. Design

To address the DoS Attacks problems discussed in the previous sections, we introduce DoSDefender, an efficient kernel-mode defense framework against SDN-aimed TCP DoS attacks implemented in the data plane. DoSDefender prevents TCP DoS attacks from entering SDN and effectively handle TCP DoS attacks in low computing consumption while maintaining low connection delay and high packet forwarding throughput. The detailed design of DoSDefender in Open vSwitch, commonly used as the data plane, is presented in this section.

### 4.1. System Architecture

**Figure 2:**
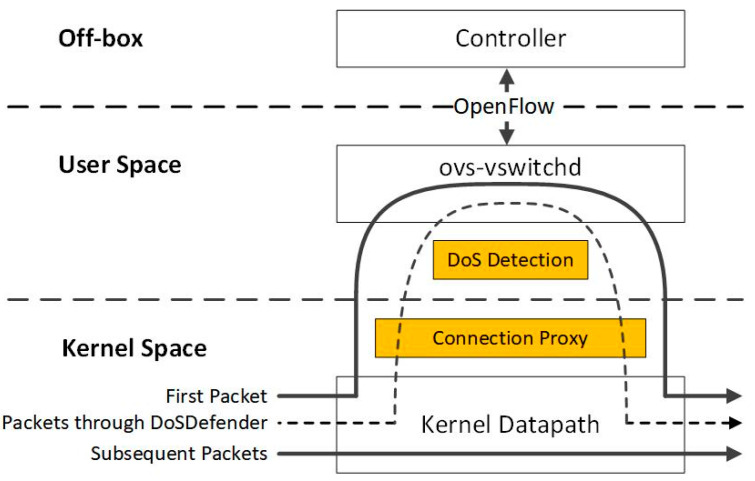
The components of DoSDefender in Open vSwitch.

DoSDefender extends the data plane with two additional components: (1) DoS detection and (2) connection proxy. Figure 2 depicts how the two functional components of DoSDefender work together to handle packets with the ovs-vswitchd and kernel datapath of the main Open vSwitch components.

The kernel datapath in Open vSwitch receives the incoming packets from the network interface card (NIC). Then the kernel datapath matches the packet header fields and follows the cached per transport connection forwarding decisions from ovs-vswitchd in user space to handle the packets. When the first packet of a new connection comes, it is delivered to ovs-vswitchd (solid line in Figure 2). Ovs-vswitchd sends the packet-in message to the controller, receives the flow rule and the actions applied from the controller, and caches the forwarding decision for subsequent packets in the kernel datapath. Then, the subsequent packets in the normal connection will be directly forwarded (solid line in Figure 2).

DoS detection in user space polls and obtains cache traffic statistics periodically (once per second), which is used to detect DoS attacks. When a DoS attack is detected, a connection proxy in kernel space is triggered to start filtering failed TCP connections and preventing TCP DoS attacks from entering SDN in the data plane prior to delivering the packet to ovs-vswitchd (dotted line in Figure 2). Connection proxy migrates the connection and relays the packets of the valid TCP connection between the source and the destination. Connection proxy runs in kernel space since copying packets from kernel space to user space severely degrades the performance.

### 4.2. DoS Detection

DoS detection in user space polls and obtains a set of statistical traffic features periodically (once per second), e.g., packet number, packet size, flow number, and average duration per flow. DoS detection continues monitoring the network status and traffic feature for attack detection, as Q-MIND [23]. Self-organizing maps (SOM-unsupervised learning) based classifier is adopted to detect DoS attacks. DoS detection works in user space to avoid floating point arithmetic of SOM in kernel space. When DoS attacks are detected, DoS detection triggers a connection proxy into the active state. When DoS attacks are detected to be over, the connection proxy will be stopped.

### 4.3. Connection Proxy

Attackers generally flood the network by sending a massive of TCP SYN packets with forged source addresses. The forged packets do not establish a valid TCP connection but incur a massive of table-miss packets in the data plane. The table-miss will trigger a massive packet-in message from the data plane to the control plane and consume the communication bandwidth, CPU computation resource, and memory in the control plane and data plane.

The object of a connection proxy is to verify the validity of the attempts to establish a TCP connection from the source address and prevent TCP DoS attacks from entering the network. Connection proxy can differentiate the sources that will completely establish the connections from those that will not. The packets in the established connection can be permitted to enter the network. Connection proxy in the data plane migrates the TCP connection and relays the packets between the source and the destination, which includes TCP handshake, data, and FIN/RST. A connection proxy in kernel space can avoid copying packets between kernel space and user space, degrading performance when migrating connections and relaying packets.

**Figure 3:**
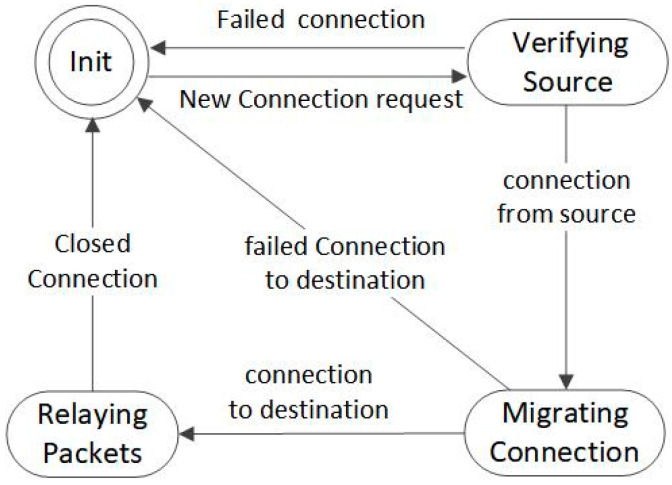
State transition of connection proxy.

Connection proxy constructs deterministic finite state automaton, which consists of four stages: (1) Initial State, (2) Verifying Source, (3) Migrating Connection, (4) Relaying Packets. The state conversions in the scheme are shown in Figure 3. When no attack is detected, the connection proxy is in a sleeping state. Once DoS attacks are detected, it changes into an active state, Initial State, and starts to mitigate DoS attacks. When the attacks are terminated, the connection proxy returns back to the sleeping state and waits to be triggered.

**Figure 4:**
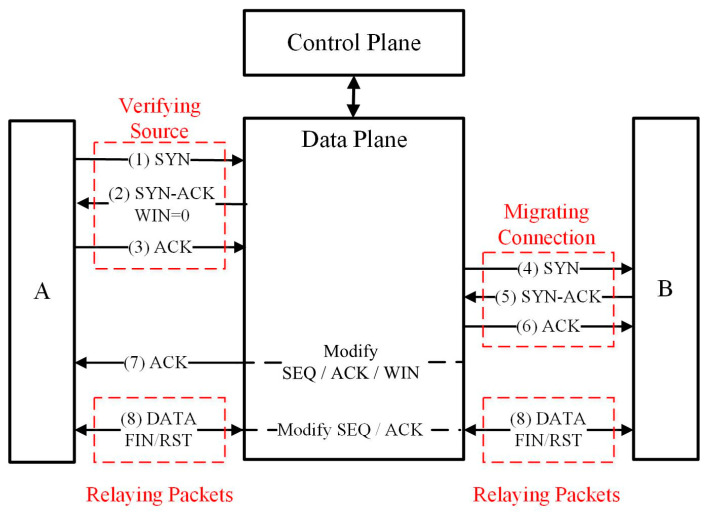
Interaction of Connection Proxy.

When an SYN packet arrives and a new TCP connection begins, the state of the connection proxy transmits from Initial State to Verifying Source, and the connection proxy verifies the validity of the connection from the source based on the completion of the TCP three-way handshake. If the connection is valid, the state of connection proxy transmits from Verifying Source to Migrating Connection. The connection proxy attempts to establish a TCP connection with the destination in the state of Migrating Connection. If the connection is established, the state of the connection proxy is transmitted to Relaying Packets. Packets are relayed between the source and the destination until the connection is closed. If the condition is not met or an exception occurs during the state transition, the state of the connection proxy will return back to Initial State. To illustrate connection proxy, consider the interaction shown in Figure 4.

(1) Initial State

When a TCP packet with SYN arrives, it means a new TCP connection. The connection proxy begins to filter each connection. The connection proxy caches the packet and searches in the cached proxy table. If it not exists, the connection proxy adds a new connection item for the packet in the proxy table. The address, sequence number, acknowledgment number, etc., of the source and the destination in the packets are filled in the new connection item of the proxy table. The state of the new item in the proxy table is set to Verifying Source, and the packet will be further processed in Verifying Source. If it exists, the packet will proceed in the current state of the corresponding connection item in the proxy table.

(2) Verifying Source

Connection proxy verifies the validity of the attempts to establish a new connection from the source, A and distinguishes the genuine attempts to establish a connection from the bogus attempts. The connection proxy pretends to be the destination, B and replies TCP packet with SYN/ACK, the second packet of a three-way handshake to A. The window size of the packet is set to zero to prevent A from sending data packets before the packet processing in the states of Verifying Source and Migrating Connection are finished. If A sends back the third packet of a three-way handshake with the right sequence number and acknowledgment number, A is identified as a normal node that wants to establish a real TCP connection and not a DoS attacker. The state of the connection proxy will be transmitted to Migrating Connection. Otherwise, if A is identified as an abnormal node, the packets will be dropped.

(3) Migrating Connection

Migrating Connection pretends to be A and establishes a new TCP connection with B by sending a TCP SYN packet to B. When the packet with SYN/ACK from B is received, the connection proxy sends back a packet with ACK. Once the proxy connection with B is established, the connection proxy sends A a packet with the right acknowledgment number, sequence number, and normal window size. Then, A can send data packets. The state of the corresponding connection proxy is transmitted from Migrating Connection to Relaying Packets.

(4) Relaying Packets

When a new incoming packet arrives, it will be searched in the proxy table. If matched, the packets with the modified sequence number and acknowledgment number will be forwarded to the peer. When the packet with RST/FIN arrives, it means that the TCP connection needs to be closed, and the responding connection item of the packet will be removed from the proxy table.

## 5. Evaluation

**Figure 5:**
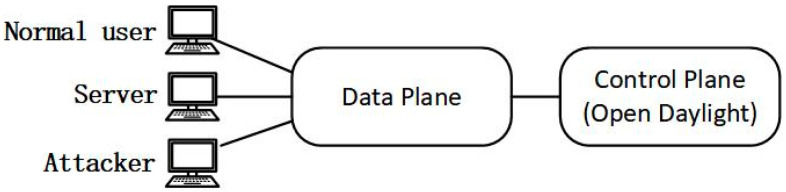
Environment for DoS attack scenario.

DoSDefender is implemented in the data plane to evaluate the effect for preventing TCP DoS attacks in the de facto standard SDN, OpenFlow network. The experiment environment for preventing TCP DoS attacks is depicted in Figure 5. Three hosts with Intel I350 NIC are used as the normal user, attacker, and server. OpenDaylight Beryllium controller is used as the control plane on a computer.

To evaluate DoSDefender, we launch the SDN-aimed TCP DoS attacks in three scenarios: (i) data plane with Open vSwitch, (ii) data plane with Avant-Guard, and (iii) data plane with DoSDefender. Iperf3 is used to generate normal traffic and measure the available bandwidth from the normal user to the server. NetSniff-ng is used by the attacker to generate TCP DoS attacks against the server.

### 5.1. Preventing TCP DoS Attacks

**Figure 6:**
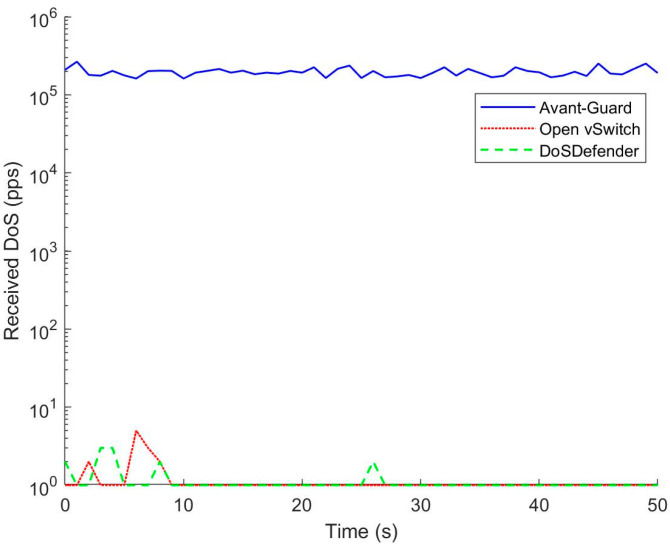
Preventing TCP DoS attacks.

The attacker launches 1 Mpps TCP DoS attack on the server. The rate of DoS packets received by the server in three scenarios is depicted in Figure 6. The average rate of DoS delivered to the server is 227 Kpps in the OpenFlow network with Open vSwitch. The intensity of TCP DoS attacks exceeds the processing capability of the controller, and most of the DoS packets are lost. In contrast, DoS attack packets are not delivered to the server with Avant-Guard and DoSDefender. Both Avant-Guard and DoSDefender can efficiently protect the OpenFlow network.

### 5.2. Computation Resource Consumption

**Figure 7:**
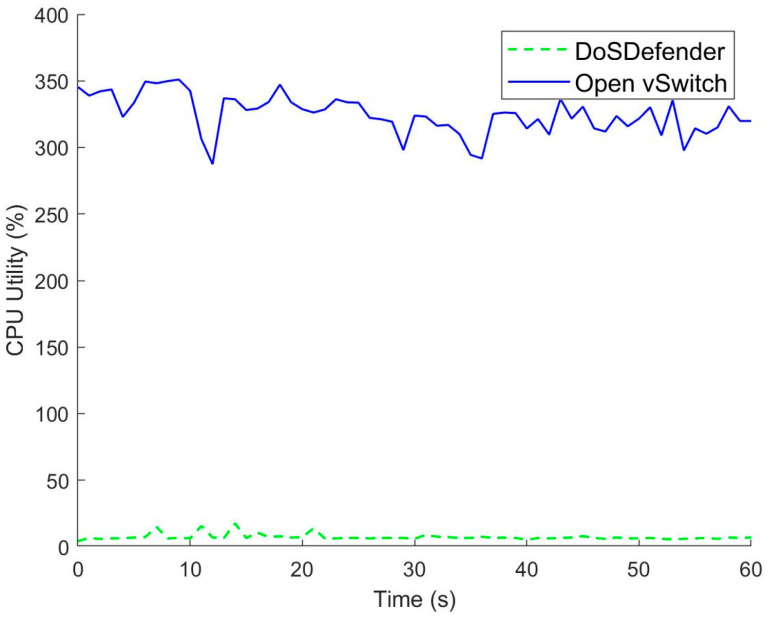
CPU Utility.

The attacker launches 1 Mpps TCP DoS traffic in the experiment. The controller’s CPU utility is depicted in Figure 7. The average CPU utilization of the controller is 324% in the OpenFlow network with Open vSwitch. TCP DoS attacks incur a massive table miss in the data plane. The data plane sends the controller a massive of packet-in messages, which heavily consume the computation resource of the controller. DoS attack packets are not delivered to the server with protecting mechanism (i.e., DoSDefender or Avant-Guard), and the average CPU utilization of the controller is less than 7.3%. DoSDefender can protect the computation resource of the controller against TCP DoS attacks.

### 5.3. Delay in Establishing a Connection

**Figure 8:**
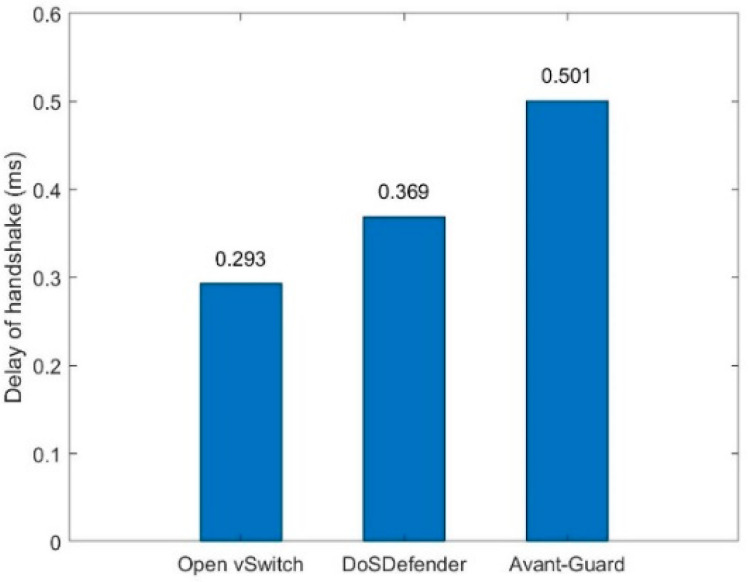
Delay of establishing connection.

The average delay in establishing a connection that finishes a three-way handshake in three scenarios is depicted in Figure 8. The average delay of establishing a connection with DoSDefender is more about 0.07 ms than with Open vSwitch because DoSDefender inevitably consumes extra time verifying source and migrating connection. The average delay of establishing a connection with DoSDefender is less than 0.14 ms than that with Avant-Guard because there is an overhead of copying packets from kernel space to user space in the slow userspace datapath with Avant-Guard.

### 5.4. Available Bandwidth

**Figure 9:**
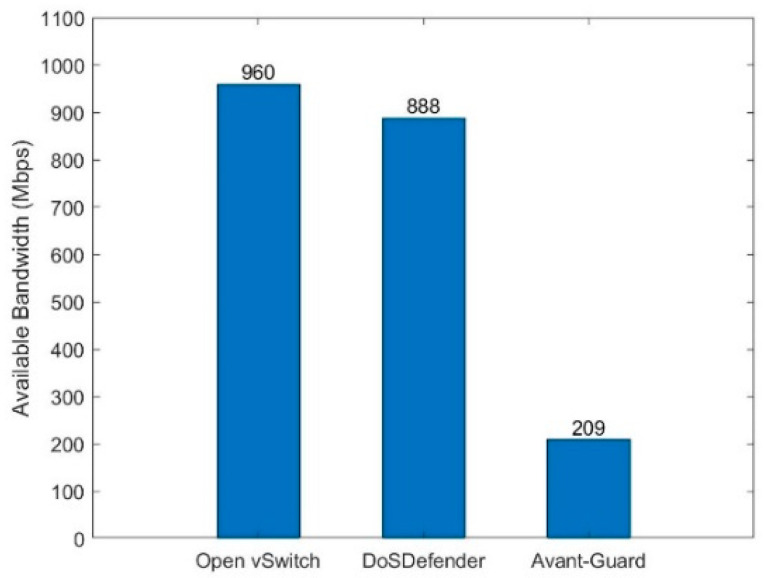
Available bandwidth.

Normal traffic is generated to measure the available bandwidth between the normal user and the server. The available bandwidth in three scenarios is depicted in Figure 9. The available bandwidth with DoSDefender is about 888 Mbps, less than 7.5% than 960 Mbps with Open vSwitch, because DoSDefender needs to finish extra work verifying the source and migrating the connection, etc. The available bandwidth with DoSDefender is more than that with Avant-Guard since Avant-Guard needs to copy packets from kernel space to user space in the relaying stage.

## 6. Conclusions and Future Work

TCP DoS attacks significantly degrade the performance of SDN and consume the resources of the control plane by leveraging a reactive centralized packet processing mechanism with the match-action paradigm. To mitigate this security threat, we introduce DoSDefender, an efficient kernel-mode TCP DoS prevention framework in the data plane to prevent TCP DoS attacks from entering SDN. DoSDefender monitors the ingress traffic in the data plane and detects DoS attacks. In the TCP DoS attacks case, every packet with TCP SYN arrives; DoSDefender verifies the validity of the attempts to establish a new TCP connection from the source. The TCP packets of the valid flow can be forwarded, and those of the invalid flow will be dropped. DoSDefender migrates the connection and relays the packets between the source and the destination in kernel space which can avoid the extra overhead of copying packets between kernel space and user space and degrade the performance. Compared with previous work, DoSDefender can effectively prevent TCP DoS attacks and protect the controller’s computation resource while maintaining low connection delay and high packet forwarding throughput. DoSDefender is easy to deploy without requiring no additional devices and no modification on the control plane. For future work, we will extend DoSDefender to better support more sophisticated attacks and non-TCP DoS attacks which are based on UDP or ICMP protocols.

## Data Availability

Not applicable.
